# Urban survival, population dynamics and historical transitions in Thessaloniki (316 BC-AD 1500)

**DOI:** 10.1007/s12520-026-02439-z

**Published:** 2026-03-18

**Authors:** Asterios Aidonis, Christina Kakasa, Elissavet Ganiatsou, Panagiota Bantavanou, Soultana Protopsalti, Stavroula Tzevreni, Stela Vasileiadou, Krino Konstantinidou, Christina Papageorgopoulou

**Affiliations:** 1 Laboratory of Biological Anthropology, Department of Humanities, Democritus University of Thrace, Komotini 69100, Greece; 2 Institute of Applied Bioarchaeological Research, Democritus University of Thrace, Komotini 69100, Greece; 3 Ephorate of Antiquities of Thessaloniki City, Ministry of Culture, Thessaloniki 54646, Greece

**Keywords:** Bioarchaeology, Paleodemography, Survival analysis, History of urbanization, Diachronic cities

## Abstract

Cities have historically endured profound challenges while sustaining their populations through adaptive structures and functions. Understanding contemporary urbanization requires situating it within these long-term historical trajectories. This study examines the demographic consequences of historical transitions in Thessaloniki over 1,800 years, based on 846 individuals recovered from peri-urban and intramural cemeteries. Survival analysis was applied to assess mortality risk and life expectancy across six periods: Hellenistic (323–31 BC), Roman (31 BC–AD 324), Early Byzantine (AD 324–842), Middle Byzantine (AD 842–1204), Late Byzantine (AD 1204–1430), and Post-Byzantine (AD 1430–c. 1600). The results demonstrate that from the Hellenistic through the Byzantine periods, mean survival remained largely stable, with no statistically significant fluctuations. In contrast, a pronounced decline in survival was observed during the early Post-Byzantine period, attributable to a succession of catastrophic events between AD 1422 and 1430, including a plague epidemic, an extended siege, and the city’s eventual conquest by the Ottomans. No significant differences in survival were identified between males and females, nor between individuals buried in different cemetery locations, indicating a relatively uniform distribution of urban mortality risks. By reconstructing demographic patterns across an extended temporal framework, this study highlights the interplay of biological and historical forces in shaping urban experience in Thessaloniki across nearly two millennia.

## Introduction

For most of human history, the predominant mode of habitation was settlements with low population density (Klein Goldewijk et al. 2010). Urbanization emerged as a pivotal shift (Algaze 2001; Altaweel 2019; Betsinger and DeWitte 2021; Engel and Brückner 2021; Gates 2011), and within a remarkably brief timespan in terms of evolution, urban living became the predominant form of human settlement. Today, more than half of the world’s population (55%) lives in cities, and this figure is expected to rise to 66% by 2050 (United Nations, 2018), making the study of urban demographics increasingly important in the twenty-first century. However, despite the historical increase in urbanization, past urban environments have been associated with adverse health effects and are often considered detrimental to historical populations (Christ and González Gutiérrez 2022; Crane-Kramer and Buckberry 2020; Newton and Smith 2013; Woods 2003). Empirical research has shown that city dwellers experienced an “urban mortality penalty”, mainly attributed to the prevalence of infectious diseases, periodic food shortages, and lack of adequate hygiene practices (Lewis 2002; Nagaoka et al. 2019; Olga Hernández Espinoza and Márquez Morfín 2015; Roberts and Manchester 2010; Storey 1985; Walter and DeWitte 2017).

While increased urban mortality was a reality, there are also studies indicating a significant degree of variability in the ways these processes affected urban populations (Betsinger and DeWitte 2021). In fact, during their historical transitions, cities have shown remarkable sustainability in providing continuity of habitation. Key elements that ancient cities integrated to achieve sustainability include demographic adaptability, exploitation of geographic perspectives, institution development, and culture (Smith et al. 2021). In demographic terms, it has been suggested that the viability of ancient cities was maintained through increased immigration and higher fertility rates, which countered the losses due to rising mortality rates (Bernard 2016; Bogin 1988; Dyson 1992; Killgrove and Montgomery 2016; Torres et al. 2019).

This pattern reflects a broader principle observed in pre-modern urban societies, where the dynamics of mortality, fertility, and migration were closely intertwined. In early modern European cities, for example, natural decrease was common, yet urban populations persisted or even grew due to substantial in-migration, which replenished the labor force and countered demographic losses (Galley 1995; Sharlin 1978). Similarly, studies of Roman cities show that urban population levels relied heavily on migrants from rural areas and smaller towns, as natural fertility alone could not sustain the cities in the face of high mortality, particularly among children and adolescents (Tacoma 2016).

To better understand these dynamics, the analysis of human skeletal remains provides a unique perspective on the lived experiences of past urban populations, offering critical data on their demographic trends and expanding the historical interpretations. For example, the incorporation of Britain into the Roman Empire after 54–55 BC and the introduction of urbanism, resulted in increased mortality risk for children and older men, thereby challenging the embedded belief concerning the benefits of “Romanization” (Redfern and DeWitte 2011). On the other hand, during the Medieval and Early Modern periods (AD 1100–1850), the age at death of the population of London remained relatively stable around 30 years (Müller-Scheeßel et al. 2024), while after the 17th century, life expectancy and survivorship of adults showed a marked increase, as a result of the second epidemiological transition (DeWitte 2014a; Müller-Scheeßel et al., 2024; Yaussy et al. 2023). Thus, the interplay between historical transitions and urban demography is crucial to understanding the functions that shaped the demographic patterns, and contributed to the resilience of past urban societies.

To this end, the analysis of human remains is a substantial contribution to understanding the demographic shifts that occurred following historical transitions. Current bioarchaeological studies have incorporated survival analysis, a body of statistical procedures that includes both survival functions (e.g., Kaplan–Meier estimates) and hazard models (e.g., Gompertz hazard models and Cox proportional hazards regression) (Clark et al. 2003; Kleinbaum and Klein 2012; Moore 2016), to investigate these demographic trends. For example, Gompertz hazard models have been used to explore differences in mortality risk in the Middle Cumberland Region of Tennessee during the Mississippian period (ca. 1000–1500 AD) (Fojas 2022), while Cox proportional hazards analyses have been applied to investigate the demographic collapse of Indigenous populations in California following European contact (Jones et al. 2021). Additionally, Kaplan-Meier survival analysis has been employed to investigate survivorship in industrial era London(Yaussy et al. 2023), as well as patterns of urbanization in Roman-period Austria (Sammut 2025). These approaches have been proved to be effective in describing the different trends of urbanization in the past (Betsinger et al. 2020; Betsinger and DeWitte 2017; DeWitte 2024; Walter and DeWitte 2017; White et al. 2022). However, rarely have been employed in the context of the circum-Mediterranean region (Biehler-Gomez et al. 2024), where a series of diachronic cities, such as Thessaloniki, Ephesus, Jerusalem, Marseille, or Cordoba, share much in common regarding their urban patterns and historical transitions (Harper 2017).

The present study explores how the synergistic impact of historical phenomena and urbanization affected the population of Thessaloniki. The city of Thessaloniki is a European urban hot spot in the eastern Mediterranean and a representative example of uninterrupted urban development, with a historical course that provides a unique record of transitions, invasions, conquests and epidemic outbreaks. In recent years, large-scale excavations for the metropolitan subway construction unearthed more than 4,000 graves from peri-urban cemeteries of the city (Lambrothanassi et al. 2018). This bioarchaeological archive, dated from the 3rd century BC to the 15th century AD represents the lived history of the population, and provides significant biocultural data for understanding the challenges of urban life.

In this perspective, we evaluated the impact of Thessaloniki’s historical transitions on the population. We studied 664 individuals to reconstruct the city’s demographic trends, in the Hellenistic (323 − 31 BC), Roman (31 BC – AD 324), Early (AD 324–842), Middle (AD 842–1204), Late (AD 1204–1430) and Post Byzantine (AD 1430 - c. 1600) periods, and contextualized the findings with the city’s historical trajectory. As transitional periods with historical complexity and social instability are often related to fluctuation in living conditions, we examined whether these transformations were associated with changes in mortality risk and survival patterns of the population.

## Materials and methods

### Historical background and cultural context

Thessaloniki was founded by Cassander in 316/5 BC through the amalgamation of 26 surrounding towns and villages. It was named after his wife, Thessaloniki, the daughter of Philip II and half-sister of Alexander the Great. The city became a pivotal economic and political hub due to its strategic position along the trade routes that connected the Aegean Sea with the Balkan hinterland. After 168 BC, under Roman rule, Thessaloniki was designated as the capital of *Provincia Macedonia*, leading to a significant influx of diverse populations (Antonaras 2019; Nigdelis 2010; Vanderspoel 2010) and fostering urban growth, evidenced by increased residential construction between the 2nd and 4th centuries AD (Salomies 1996; Stefanidou-Tiveriou 2010; Vitti 1996).

Following the establishment of the Byzantine Empire, Thessaloniki retained its political and economic status. The advent of Christianity and construction of numerous churches, many of which have been designated as UNESCO World Heritage Monuments, profoundly influenced both the urban and social topography of the city (Drakoulis 2012). Until the 7th century AD, the city faced repeated raids by the Goths, Huns and Avars, and experienced the outbreak of the Justinian plague (Laiou and Morrisson 2007; Stathakopoulos 2000). The subsequent Slavic invasions led many people to migrate to Thessaloniki and its surroundings in search of refuge (Antonaras 2019). In the next centuries, despite being sacked by Saracens (9th century), engaged in conflicts with the Bulgarians, and undergone a second sack by the Normans in AD 1185, the city remained a military, administrative, and commercial center, exerting strong ideological and cultural influence in the Balkans (Kourkoutidou-Nikolaidou and Tourta 1997; Treadgold 1997). After Constantinople was captured by the Crusaders, leading to political fragmentation of the empire, Thessaloniki became the capital of the short-lived Frankish kingdom (AD 1204–1224). In the second half of the 14th and the early 15th century, recurring plague epidemics, internal strife, and wars caused population decline and eventually the city was occupied by the Turks in 1430 (Laiou and Morrisson 2007).

Summarizing the historical course, four key phases of the city’s development can be distinguished: (a) its establishment during the Hellenistic period, (b) the intensification of urbanization during Roman times, which led to expansion of the inhabited area and the population, (c) the city’s resilience during the turbulent Byzantine era, marked by repeated invasions and epidemics, and (d) the period immediately following its capture by the Ottomans. These historical transitions have been extensively studied from archaeological and historical perspective, but little is known about their impact on the everyday life and demography of the city’s inhabitants.

### The skeletal material

**Fig. 1:**
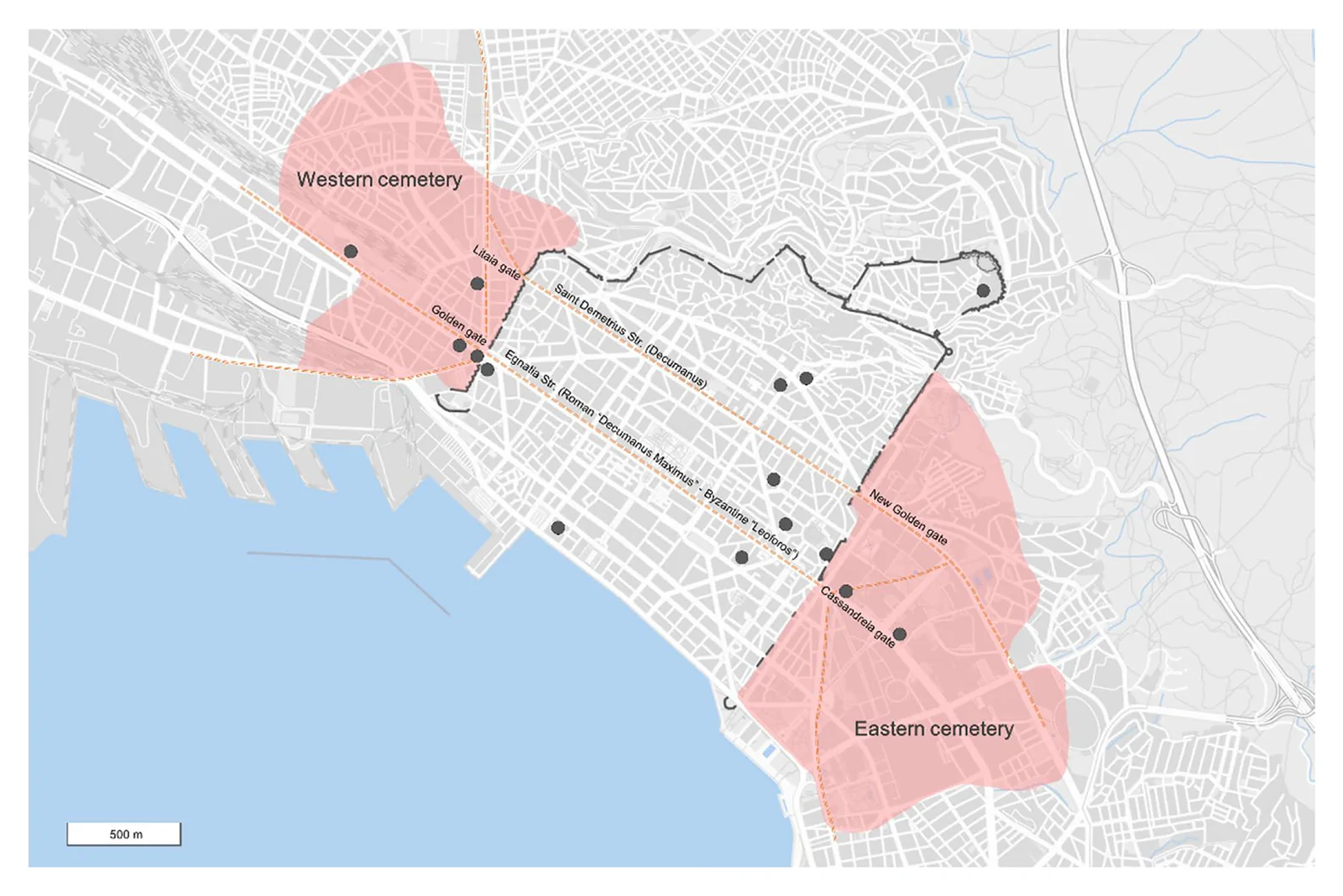
Map of Thessaloniki depicting the eastern and western cemeteries (shaded red), located outside the city walls (outlined in grey), along with the excavation sites (black dots) representing the origin of the skeletal material. The orange dotted lines represent the roads leading to the main gates of the ancient city, many of which remain in use today (modified from Marki 2006)

Throughout the Hellenistic, Roman, and Early Byzantine periods, the cemeteries of Thessaloniki extended outside the walls, along the roads leading to the main gates (Fig. 1), forming the eastern and western *necropoleis* of the city (Marki 2006). Around the 7th century AD, cemeteries outside the walls ceased to be used systematically and burials appeared around churches and monasteries within the city walls (Bakirtzis 2003). This shift in burial practices has been attributed to a change in the conceptualization of *ad sanctos* burials and urban space as well as the insecurity caused by frequent raids (Chatzinikolaou and Terzopoulou 2012; Wataghin 1999). In total, 846 individuals were studied from the east and west peri-urban necropoleis as well as intramural cemeteries of Thessaloniki. However, data from 182 skeletons were excluded from the analysis owing to the absence of secure chronological attribution, resulting in a final sample of 664 individuals. Chronological assignments of skeletons were primarily based on artifact associations and stratigraphic position. Burials that could not be assigned to one of the aforementioned periods were classified as undated. The material under study has been excavated by the Ephorate of Antiquities of the City of Thessaloniki, mainly during the large-scale works for the construction of the metropolitan subway. The skeletal analysis was conducted in accordance with permissions granted by the Ephorate of Antiquities of the City of Thessaloniki, the regional service of the Ministry of Culture responsible for the protection and scientific research of ancient cultural heritage.

### Age and sex estimation

Age estimation for non-adults was based on tooth development, diaphyseal length and epiphyseal closure of long bones (Buikstra and Ubelaker 1994; Cunningham et al. 2016; Ferembach et al. 1980). Adult age was estimated based on age-related changes in the pubic symphysis, auricular surface, cranial suture closure and dental wear (Brooks and Suchey 1990; Brothwell 1981; Lovejoy et al. 1985; Meindl and Lovejoy 1985). The skeletons were assigned to one of the following age groups: Fetal (< birth), Infant (0 to 3 year), Child (4 to 12 year), Adolescent (13 to 18 year), Young Adult (19 to 34 year), Middle Adult (35 to 49 year), Old Adult (50 + yr). Individuals lacking precise age markers were grouped in broader categories (nonadult/adult). Sex was estimated using standard methods based on pelvic and cranial morphological variation (Bass 2005; Buikstra and Ubelaker 1994; Ferembach et al. 1980; Phenice 1969). The skeletons were initially categorized as female, probably female, indeterminate, probably male, or male. However, following standard bioarchaeological practice, individuals were grouped into three categories for statistical analysis: male, female, and indeterminate (which included nonadults, individuals with non-diagnostic features, or those with poor skeletal preservation).

### Demographic analysis

A comparison of survivorship patterns over time was conducted based on age-at-death estimates derived from skeletal remains. All the analyses were run in R and SPSS. The mortAAR package (Mueller-Scheessel et al. 2025) was used to evaluate the representativeness of the nonadults in the dataset. The relevant function of the mortAAR package uses indices to compare the proportions observed among nonadult age groups with those documented in contemporary, comparable populations (Bocquet-Appel and Masset 1977; Taylor and Oxenham 2025; Weiss and Wobst 1973). The impact of bone preservation on the representativeness of the sample was investigated by calculating the preservation index following the method proposed by Stojanowski et al. (2002). Additionally, the representativeness of the sexes within the dataset was ascertained by calculating the sex ratio $\left(SR=100*\frac{\;\text{males}\;}{\;\text{females}\;}\right)$ of the skeletal sample.

We then constructed life tables using the relevant function of the mortAAR package, which employs a statistical approach that assumes a uniform probability of individuals being distributed across the five-year age classes of the initial broader age-at-death estimations. An individual aged within the age range of 20 to 34 years was distributed by adding 1/3 to each of the three adjusted age classes. In cases of broader age estimation, smaller fractions were added to the relevant age classes (Acsadi and Nemeskeri 1970). For old adult individuals, whose maximal age is in fact open-ended, we assumed an upper limit of 80 years.

At this step we excluded the nonadults aged 0–9 years to address the observed underrepresentation of infants and children commonly present in archaeological skeletal populations (Jones et al. 2021; Kelmelis and DeWitte 2021), as well as the adults of unknown age and sex, to avoid the potential bias in the results. We then used the median age of each five-year class above 10, repeated according to the number of deaths in each class, to build the age distributions for comparing age-at-death patterns and conducting the survival analysis.

Mann–Whitney U tests were first employed to assess whether significant differences existed in age-at-death distributions between periods (Chamberlain 2006). The survival package (Therneau 2024; Therneau and Grambsch 2000) was then used to perform a Cox proportional hazards analysis in order to evaluate variations in mortality risk. We used the defined historical periods, the sex and the cemetery as covariates that may influence survival. The proportional hazards assumption for each covariate used in the model was checked with the relevant function of the survival package. Finally, we conduct a series of Kaplan–Meier analyses (Kaplan and Meier 1958) with the associated log-rank tests to investigate survivorship across and within the time periods under study and between females and males. Assuming that the eastern cemetery might represent higher status burials due to its proximity to the Galerius Palace Complex, we also examined survivorship between the burials in the city’s eastern and western cemetery.

It has been discussed by previous studies that age-at-death distributions in ancient cemeteries may better reflect fertility than mortality. Research has demonstrated that there is a strong correlation between the proportion of juveniles in cemetery samples and fertility of the once living population (Bocquet-Appel 2002; Bocquet-Appel & Naji, 2006; Buikstra et al. 1986; McFadden and Oxenham 2018; Paine 1989; Sattenspiel and Harpending 1983). Actually, an increase in fertility results in a higher proportion of nonadults and a lower mean age at death. Conversely, a decrease in fertility leads to a higher proportion of adults and a higher mean age at death (Betsinger et al. 2020; Betsinger and DeWitte 2017; Jones et al. 2021; Zoeller et al. 2022). A number of fertility proxies have been developed (Bocquet-Appel, 2002; Buikstra et al. 1986; McFadden and Oxenham 2018), and have been used to evaluate if the observed changes in survival actually represent changes in fertility (Walter and DeWitte 2017; Zoeller et al. 2022). To evaluate the effect of fertility on survivorship in the chronological and cultural periods under study, we employed the P indicator (*D*_5−19/_/*D*_5+_ ratio), which is positively correlated to birth and growth rates (Bocquet-Appel, 2002; Bocquet-Appel & Naji, 2006). In order to evaluate the significance of diachronic changes between the periods, we used the 95% comparison intervals as proposed by Buikstra (Buikstra et al. 1986).

$$CI=\frac{D30_{+}}{D5_{+}}\pm m_{05,k*,\infty }\sqrt{\frac{.125}{D5_{+}}}$$

where *m* is read from a table of the studentized maximum modulus, at a two-tailed alpha level of 0.05, with k* as the *(N(N− 1)/2* for *N* samples, and under infinite (∞) degrees of freedom (Stoline and Ury 1979). Furthermore, it has been suggested that when the population growth rate (r) is low, the P indicator provides a direct measure of mortality (Barbiera et al. 2018). Accordingly, for growth rates deviating from ± 3‰, we adjusted the P values to account for shifts in the population’s age structure resulting from changes in birth rates, using the d* indicator as described by Barbiera et al. (2018).

## Results

Nonadults under the age of ten were found to be under-represented in the Hellenistic, Early Byzantine and Post Byzantine data sets, while the Roman, Middle and Late Byzantine samples could be characterized representative, by satisfying one of the conditions either of Weiss and Wobst (1973) or Bocquet-Appel and Masset (1977). However, none of the subsamples meet the criterion of Taylor and Oxenham (2025) (see Table S1). As for bone preservation, age was found to affect it significantly (*p* < 0.001); infants aged 0–3 years (IS = 0.0831) and children aged 3–12 years (IS = 0.0883) present the lowest bone preservation, with skeletal completeness of 8.31% and 8.83% respectively (Table S2-S3). Owing to this underrepresentation, the 0–9 age classes were excluded from both the comparison of age-at-death distributions and the survival analysis.

**Fig. 2:**
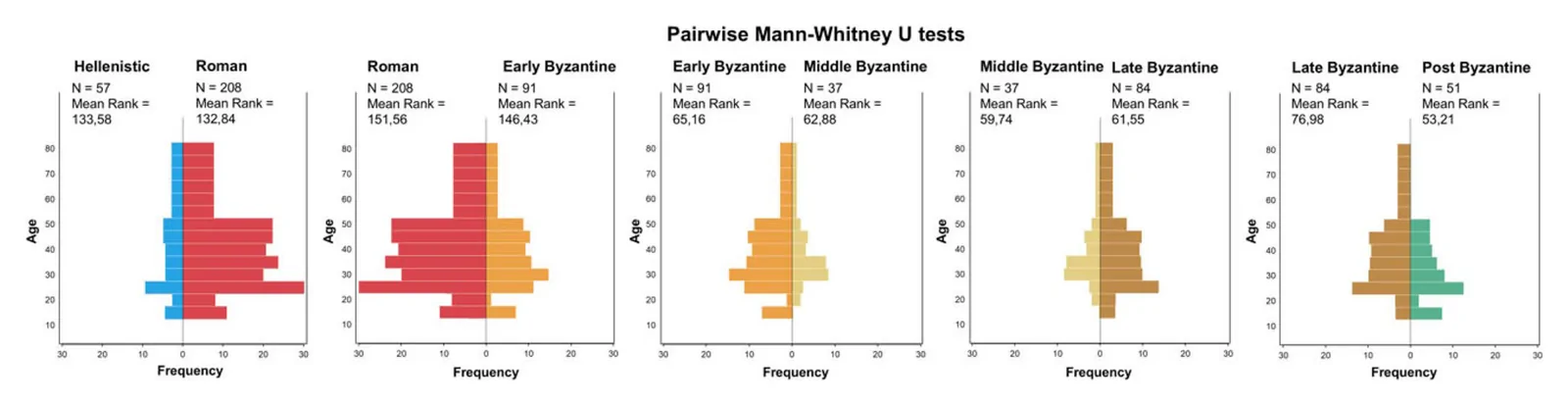
Visual representation of the Mann-Whitney U test results, illustrating age-at-death distributions across groups from the Hellenistic to the Post-Byzantine period

The age-at-death distributions for each chronological period are shown in Table 1, while the age at death distributions in 5-year age classes are presented in the form of life tables in Table S4. Life expectancy at age twenty (e20) declines from 24.4 years in the Hellenistic period to 21.86 years in the Roman period. It remains relatively stable from the Early Byzantine (20.34 years) to the Late Byzantine period (20.85 years), before dropping sharply in the Post Byzantine period to its lowest value of 12.65 years. The comparison of age-at-death distributions using the Mann-Whitney U test revealed that there were no significant differences between the Hellenistic, Roman and all three Byzantine groups. However, the Post Byzantine group did show a significant differentiation (*p* = 0.001) as depicted in Fig. 2 and Table S5. The examination of the sex ratio by chronological period indicates that females outnumber males in the Hellenistic (SR = 70.0), Roman (SR = 88.9), Early (SR = 68.6) and Middle Byzantine (SR = 52.6) periods. Conversely, there is a skew towards males in the Late Byzantine (SR = 136.4) and even more in the Post Byzantine period (SR = 241.7), where the underrepresentation of women is significant (Table S6; *p* = 0.012).

The results of the Cox proportional hazards model indicate that the covariate of period is significantly related to elevated risk of death (*p* < 0.001), while sex and burial topography are not (Table 2). Overall, the p-values for all three tests (likelihood, Wald, and score) indicate that the model is significant. When testing the proportionality assumption, there is no evidence to suggest that the proportional hazards assumption is violated, either for individual variables or globally, as all the covariates are not significant (Table S7).

**Fig. 3:**
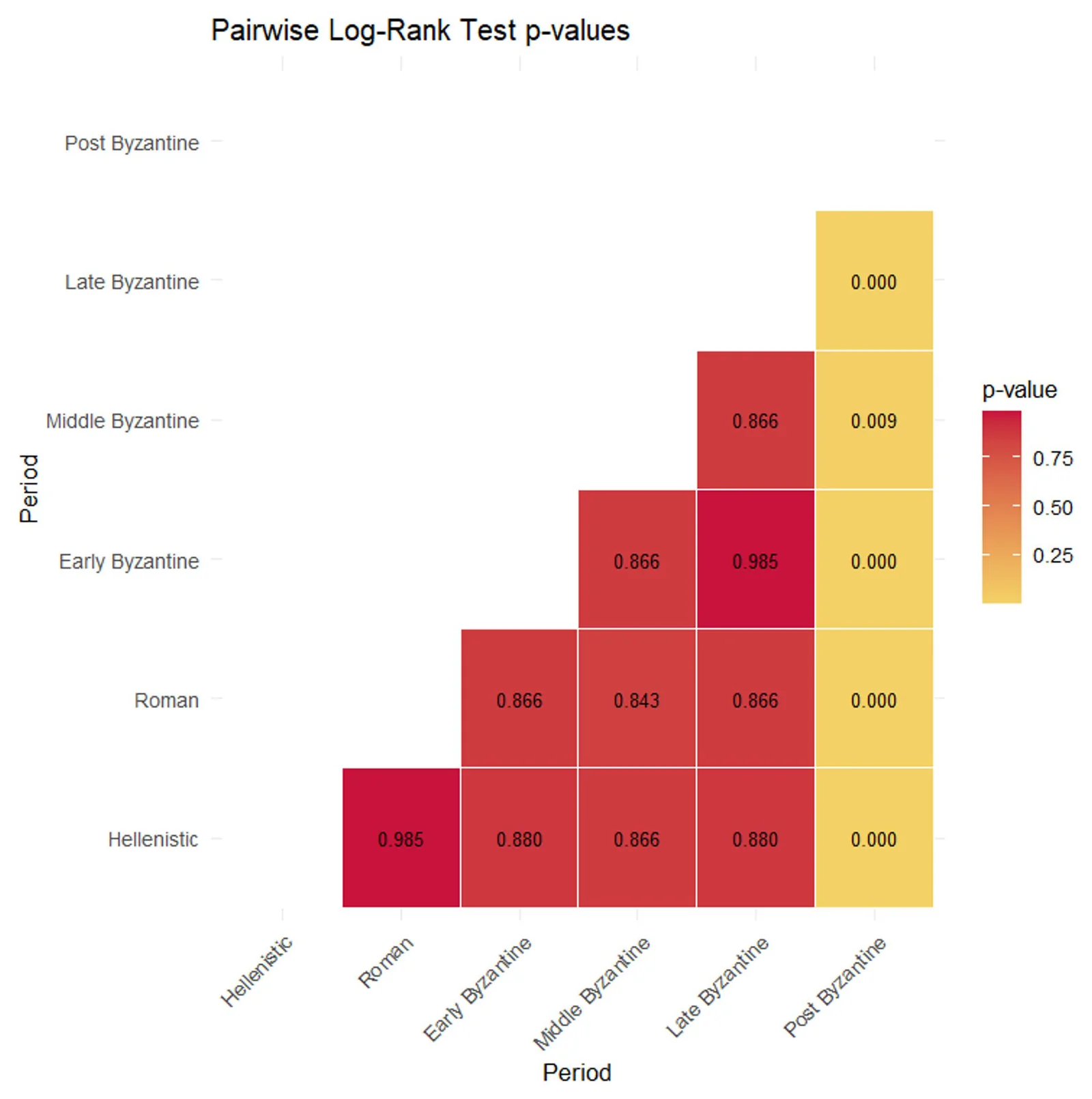
Heatmap plot showing the p-values from the pairwise Kaplan Meier comparisons between the chronological groups. Color intensity reflects the p-value, with lighter shades indicating significant differences (*p* < 0.05)

**Fig. 4:**
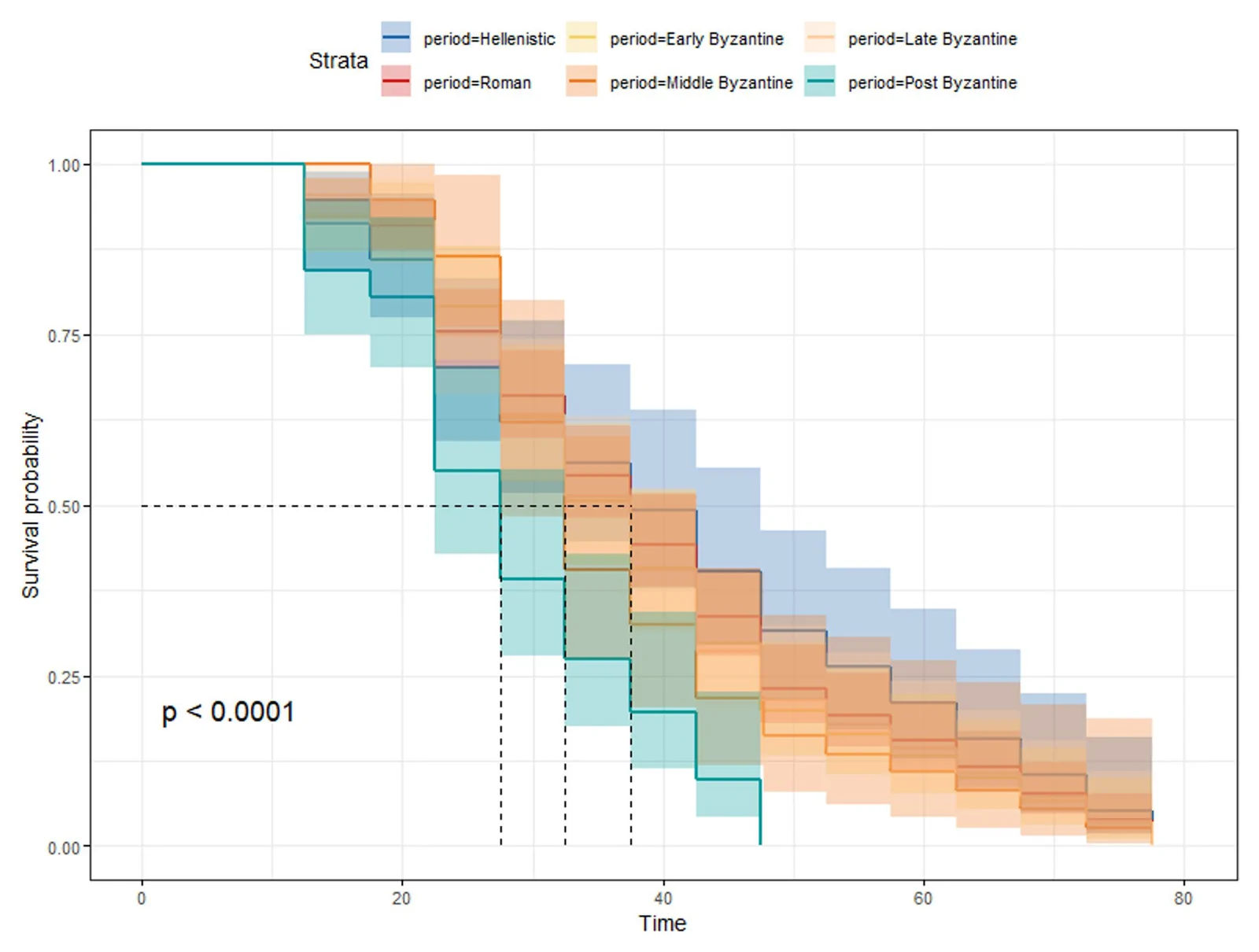
Kaplan–Meier survival curves with 95% confidence intervals indicate a significantly lower survivorship in the Post Byzantine sample

The results of the Kaplan-Meier analyses revealed difference in survivorship over time (Table 3, *p* < 0.001). Pairwise comparisons of the chronological samples indicate broadly comparable survival times from the Hellenistic through the Late Byzantine periods, suggesting demographic stability across these centuries. In contrast, the sharp decline documented in the Post Byzantine period is statistically significant, marking a clear disruption in population survival patterns (Fig. 3). This is also visually evident in the survival curves, which display reduced survivorship among Post Byzantine individuals **(**Fig. 4).

**Fig. 5:**
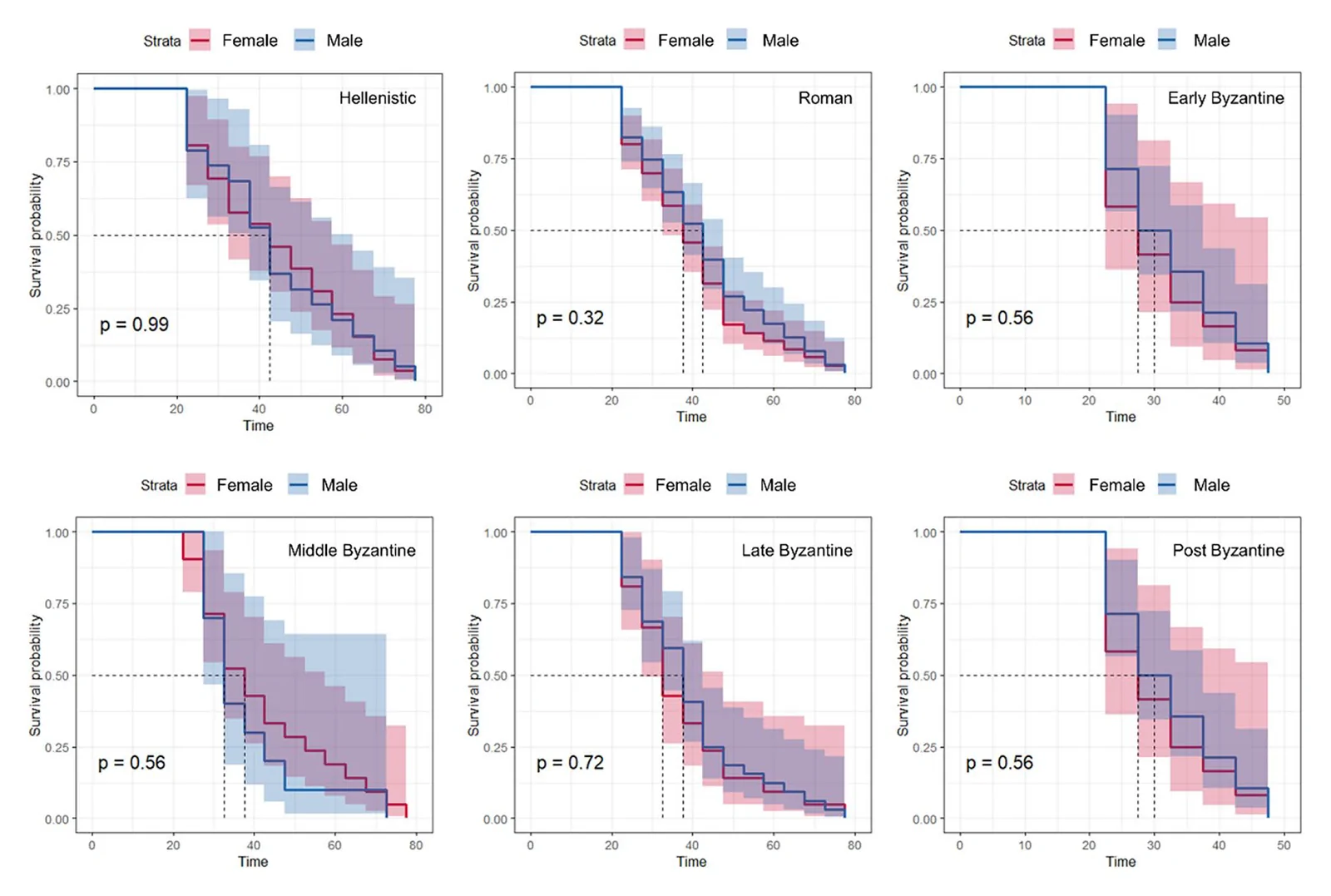
Kaplan–Meier survival curves illustrating female and male survivorship across chronological periods in Thessaloniki

**Fig. 6:**
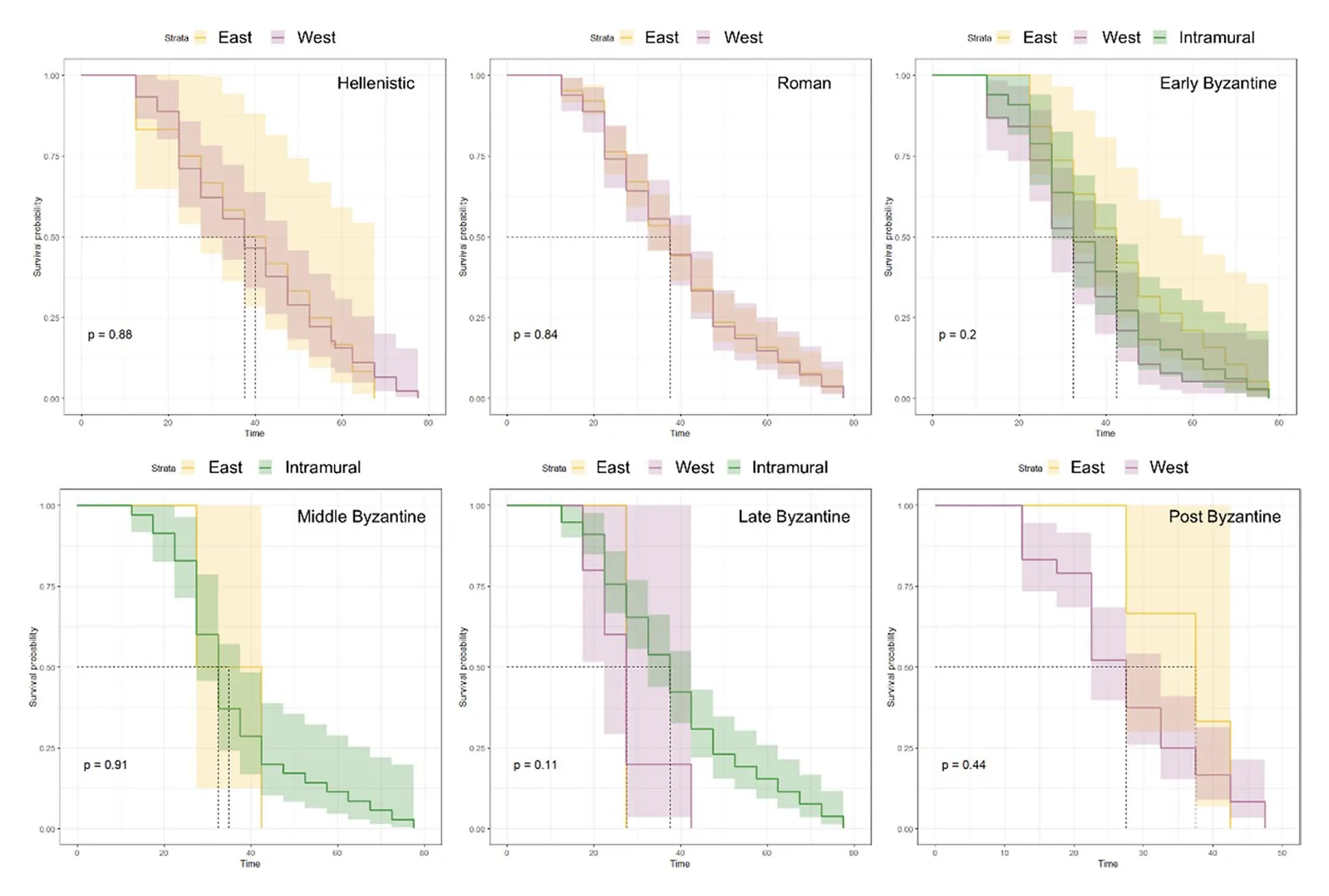
Kaplan–Meier survival curves illustrating survivorship across east, west, and intramural cemeteries by time period in Thessaloniki

**Fig. 7:**
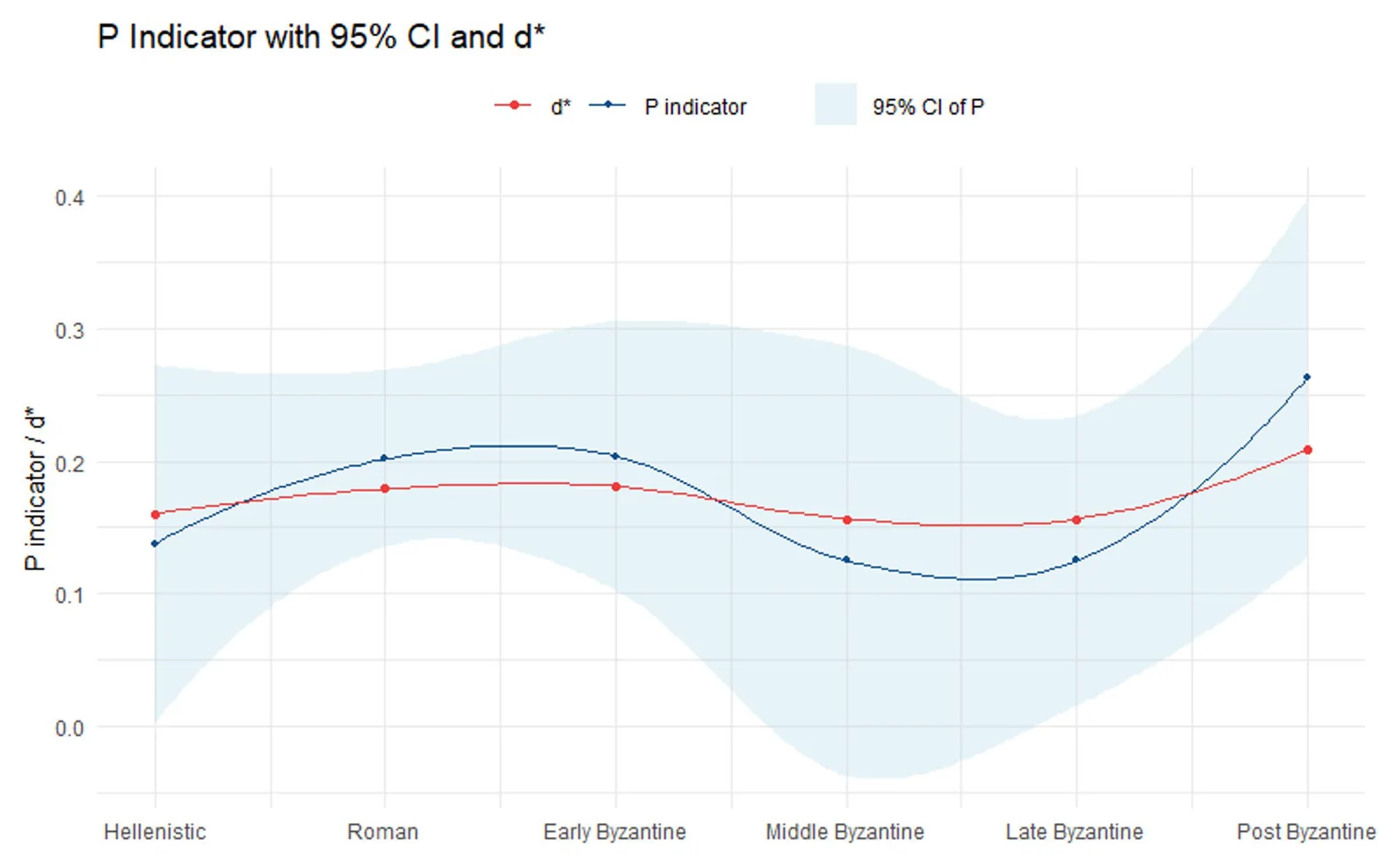
The P indicator (blue line) with 95% comparison intervals (light blue shaded area) and the r* indicator (red line) by chronological period in Thessaloniki

A second series of Kaplan-Meier analyses was performed to examine survival patterns by sex across the chronological periods. The results indicate that male and female mean survival times were broadly comparable in all periods, with no statistically significant differences (Fig. 5 and Table S8). The survival times between the eastern, western, and intramural cemeteries by period, also show no significant differences (Fig. 6 and Table S9). The P indicator (D5-19/D5+) shows fluctuations across chronological periods, starting at 0.1379 in the Hellenistic period, rising to 0.2017 in the Roman and 0.2039 in the Early Byzantine periods, then declining to 0.1250 in both the Middle and Late Byzantine periods. In the Post Byzantine period, the ratio increased markedly to 0.2632. However, the overlapping 95% comparison intervals suggest that these differences are not statistically significant. The application of the d* indicator following Barbiera et al. (2018), further reduces these variations, resulting in a smoother demographic profile across the examined periods. Across chronological periods, the r* indicator measured 0.1604 in the Hellenistic, 0.1792 in the Roman, 0.1814 in the Early Byzantine, 0.1565 in both the Middle and Late Byzantine, and 0.2092 in the Post Byzantine period. (Fig. 7; Table S10).

## Discussion

The living experience of city dwellers is shaped by the cumulative effects of historical transitions on urban infra-structure and the social fabric. The present study employed a paleodemographic analysis to examine the impact of historical processes on the demographic profile of Thessaloniki during the Hellenistic, Roman, Byzantine and Post Byzantine periods.

The analysis of bone preservation in the sample of Thessaloniki revealed that poor preservation may have affected representation. This phenomenon was observed in all chronological periods except the Roman. However, this is a common finding in bioarchaeological research and is usually attributed to: (a) cultural beliefs influencing burial practices, (b) differential preservation of the fragile infant bones, and (c) limited archaeological research (Saunders and Barrans 1999). Furthermore, children were frequently excluded from formal burial sites during Greek antiquity (Dimakis 2021; Lagia 2007) due to their late status recognition (Lagia 2007; Lancy 2014). Atypical funerary treatment of infants can be traced back to the Mycenaean period (Lebegyev 2009) and has been detected in Mendi, Halkidiki (Vokotopoulou 1989), the Agora of Athens in the so-called “well of the babies” (Liston and Rotroff 2013), and in Kylindra, Astypalaia (Hillson 2009). Although a comparable funerary treatment has yet to be identified in Thessaloniki, it cannot be dismissed. It can therefore be posited that the underrepresentation of infants and children in the Hellenistic, Byzantine and Post Byzantine sample from Thessaloniki may be influenced by cultural and taphonomic determinants, as well as the limited nature of the excavations.

The sex ratios observed in Thessaloniki vary across the different periods, but it is only in the Post Byzantine period women are significantly underrepresented. Most bioarchaeological studies indicate a systematic bias favoring males, which can be attributed to burial practices, biased methods of sex estimation, and the better preservation of male skeletons (Bennike 1985; Biehler-Gomez et al. 2022; Cintas-Peña and Herrero-Corral 2020; Weiss 1972). However, in demographic terms, the sex ratio is influenced by births, deaths, and migration (Hesketh and Xing 2006). Urban migration is often considered primarily labor migration, which depends on the availability of employment opportunities. For example, in Rome, most jobs were in manual labor and predominantly occupied by male workers (Erdkamp 2008), although alternative perspectives are also being explored (Prowse 2016). In Post Byzantine Thessaloniki, the marked underrepresentation of females is hardly explained solely by the demands of the city’s labor market. It is more reasonable to interpret this phenomenon within the broader context of the demographic upheavals between 1422 and 1430, a period marked by a plague outbreak, prolonged siege conditions, and ultimately the Ottoman conquest. The depopulation of Thessaloniki during the Ottoman siege, followed by the forced resettlement of new populations after the city’s conquest, may triggered substantial demographic restructuring. This process likely produced a male-biased population structure, which shaped both by the predominance of men among the city’s defenders and by the composition of incoming groups, many of whom were recruited for military service and other labor-intensive activities traditionally dominated by males.

The results of the Cox proportional hazards analysis indicate that chronological period is a significant predictor of mortality risk, highlighting temporal shifts in population survival. Pairwise Kaplan–Meier comparisons show no statistically significant differences in survival times between the Hellenistic, Roman, and all Byzantine periods, suggesting relative stability in mortality patterns over this extended timeframe. In contrast, the Post Byzantine sample exhibits a marked and statistically significant decline in survival. The P indicator (*D*_5−19/_/*D*_5+_) reflects temporal variation, with higher values in the Roman, Early Byzantine, and particularly the Post Byzantine period. Nevertheless, overlapping comparison intervals suggest that these differences are not statistically significant and thus do not affect survival. Beyond reflecting birth and growth rates (Bocquet-Appel 2002; Bocquet-Appel & Naji, 2006), in the case of stable populations (r = ± 3‰), this measure can serve as a direct proxy for mortality (Barbiera et al. 2018). However, the assumption of population stability does not hold during periods of mortality crises, as deaths could rise far above normal levels and reshape demographic dynamics (Barbiera et al. 2018). In Thessaloniki, the growth rate calculated using the P indicator (Bocquet-Appel, 2002) fluctuates above the ± 3‰ threshold (Table S10). When adjusted using the d* indicator (Barbiera et al. 2018), the mortality pattern mirrors the overall trend of the P indicator with higher values also in the Roman, Early Byzantine, and Post Byzantine period, but shows a much smoother distribution across periods, with less pronounced fluctuations.

An examination of life expectancy at birth (e0) reveals fluctuations between the periods under study (Table S4). It is important to note that life expectancy at birth (e_0_) in archaeological samples is strongly influenced by the systematic underrepresentation of children under five years of age, either due to differential burial practices or poor preservation of fragile remains (Chamberlain 2006; Hoppa, 2002; McFadden 2021), Indeed, such underrepresentation is evident in our skeletal sample as well. While e_0_ values are reported, they should be interpreted with caution as they primarily reflect sampling and preservation biases rather than actual population demographic conditions. For this reason, we focus our interpretation on life expectancy at age 20 (e_20_), which provides a more robust indicator less affected by these biases. Life expectancy at age 20 (e_20_) declines from the Hellenistic to the Roman period, remains relatively stable during the Byzantine period, and reaches its lowest point in the Post Byzantine period, following a pattern similar to that observed in the Kaplan Meier analysis and the P indicator.

The comparative analysis of the above demographic data along with the historical transitions that shaped Thessaloniki’s urban trajectory yields interesting insights. Archaeological sources demonstrate that during the Roman period, the economic growth and the upgrade of the city to a provincial capital resulted in a substantial population growth (Antonaras 2019; Nigdelis 2010; Vanderspoel 2010). On the basis of inhabited area, the population at this time is estimated to 70,000 inhabitants (Hanson et al. 2019), whereas the city continued to be a crowded center during the Byzantine period. Although, urban intensification has been a contributing factor to increased mortality rates, largely attributable to elevated exposure to environmental pollutants and the dissemination of infectious pathogens (Capasso 2000; DeWitte 2021; Havlíček and Morcinek 2016; Ledger et al. 2018; Mackenbach 2021; Mitchell 2017; Novak 2010; Reyes et al. 2013), our results do not support a significant correlation between the periods of intense urbanization and mortality. One possible reason for the survivorship patterns observed during these periods is the implementation of policies and legal measures aimed at improving sanitation and reducing pollution, which may have lessened some of the health risks associated with dense urban living. Archaeological and historical sources support that fundamental sanitation infrastructure (Demetriou 2002; Kaminiates 2000; Karidas 1999), such as baths (Arvanitidou 2018), and waste management mechanisms (Bakirtzis 2007; Kaiafa-Saropoulou and Karadedos 2011; Karpozilos 1989) were constructed. The residents of Thessaloniki were required to maintain the cleanliness of areas outside private property (Marki 2004), and groups of workers were responsible for supervising streets and pavements (Bakirtzis 2007). Such practices would have functioned as regulatory mechanisms for environmental pressures resulting from the negative effects of increasing population density and contributed to the resilience of the city.

Throughout the Byzantine period, the city endured the impact of the Justinianic plague, repeated sieges, looting and wars involving Saracens, Normans, Franks, and Bulgarians, as well as episodes of internal unrest such as the Zealot movement. While such crises would have inevitably impacted the population, our results show no clear evidence of survival declines during these times. Detecting the demographic impact of specific crisis events is inherently challenging, as urban populations often recovered rapidly (DeWitte 2014b; Galanaud et al. 2020; Galley 1995), and such events are more effectively investigated in catastrophic cemeteries rather than in attritional burial grounds. In Thessaloniki, despite repeated crises and episodes of expected heightened mortality, the city’s population appears to have rebounded, underscoring the resilience of urban structures. Consequently, mortality from these crises, may have caused temporary disruptions not detected in attritional cemeteries, with long-term demographic patterns largely determined by enduring urban dynamics rather than short-term shocks.

In contrast, the Post Byzantine dataset shows a marked decline in mean survival time, along with a pronounced absence of individuals over 50 years of age and a highly skewed sex ratio favoring males. This pattern may reflect the cumulative impact of a series of historical events. The Venetian occupation of Thessaloniki (AD 1423–1430), the plague outbreak of 1421–1422, the prolonged Ottoman siege after 1422, and the city’s final capture by the Ottomans in 1430 all contributed to a gradual demographic decline (Laiou and Morrisson 2007; Matschke 2002; Tsiamis et al. 2011; Vacalopoulos 1973). Following these events, Thessaloniki was nearly abandoned, with historical sources estimating the population at only around 7,000 inhabitants (Reba et al. 2016). Two years later, Ottoman authorities initiated repopulation efforts, relocating Muslims and Greeks from surrounding villages (Vacalopoulos 1973). Population displacement in the newly conquered territories was a common practice for the Ottoman authorities in order to secure the new lands and to restart the economic activity (Hooper 2003; Lowry 1986). Therefore, it is reasonable to assume that the Post Byzantine skeletal sample reflects the impact of these crises on the city’s population structure, with the significantly lower mean survival age of an immigrant population predominantly composed of young and middle adult males.

The analysis of survival across the city’s subpopulations revealed no significant differences, with males and females exhibiting comparable mean survival times across periods. In preindustrial societies, females generally exhibited lower survival rates than males, largely due to complications from pregnancy, childbirth and greater exposure to pathogens from spending more time indoors (Boldsen and Paine 1995; Cheng and Nelson 2018). By contrast, in most contemporary populations, women outlive men, a reversal known as the sex morbidity–mortality paradox. This pattern emerged in Europe only in the past few centuries, with female life expectancy first surpassing that of males in 1751 in Sweden, 1841 in England and Wales, and 1889 in Italy (Barford et al. 2006; Zarulli et al. 2018). This male excess in mortality has been attributed to the long-term demographic and epidemiological transition, with the reduction of infections and the increase of chronic diseases (Beltrán-Sánchez et al. 2015).

The bioarchaeological evidence on sex differences in mortality shows considerable variability. For instance, in Milan, females exhibited significantly lower mean survival times in Roman times, whereas no differences were observed in the Early and Late Middle Ages (Biehler-Gomez et al. 2022). Similarly, in Medieval Ireland, mortality risks did not differ between sexes (Ham 2025). In contrast, female survival rates exceeded those of males in Roman Dorset (Redfern et al. 2015). This heterogeneity likely reflects context-specific factors, which do not always align with the commonly assumed pattern of higher female mortality in preindustrial societies. The comparable mortality profiles of males and females in Thessaloniki likely reflect mortality risks that were broadly shared across the population irrespective of sex.

Additionally, the evaluation of the mean survival time between the eastern, western and the intramural cemeteries of Thessaloniki revealed also no significant differences. Previous bioarchaeological studies have demonstrated that the residents of a city may experience urban living in diverse ways and the potential benefits or disadvantages of urbanization may not distributed evenly in the population (Betsinger and DeWitte 2021; Crane-Kramer and Buckberry 2023; White et al. 2022). However, such patterns were not observed in our sample, as the current data indicate a uniform distribution of mortality risks across demographic groups in each chronological period. However, further investigation is needed, as these results may have been influenced by the limitations of the dataset, particularly the fragmentary nature of the excavations, which were often confined to restricted areas within the extensive cemeteries of Thessaloniki, and may therefore have obscured more subtle differences.

## Conclusion

This study presents a diachronic analysis of survivorship in the population of Thessaloniki, and provides informative results on the demographic impact the city experienced during its historical transitions. The findings suggest that from the Hellenistic period to the end of the Byzantine era, there were no significant changes in the average survival time. Despite the occurrence of historically documented periods of crisis, mainly during the Byzantine period, this is not reflected in the demographic patterns of the population. However, there was a statistically significant decline detected in the Post Byzantine period, showing that the city’s demographic composition changed significantly only when the population experienced the consequences of catastrophic events between 1422 and 1430. No significant difference in survival was detected between the sexes and between the individuals of the city’s cemeteries, indicating a uniform distribution of urban risks in each chronological period. Despite its limitations as every bioarchaeological study, this research contributes to a growing body of knowledge and provides a valuable record of the diachronic population dynamics of Thessaloniki by presenting a demographic chart of the city over a period of 1,800 years.

## Supplementary Material

**Supplementary Information** The online version contains supplementary material available at https://doi.org/10.1007/s12520-026-02439-z.

Supplementary Information

## Figures and Tables

**Table 1:** Distribution of individuals from Thessaloniki per age group and chronological period

	Hellenistic			Roman			Early Byzantine			Middle Byzantine			Late Byzantine			Post-Byzantine			Total	
	(323 − 31 BC)			(31 BC − AD 324)			(AD 324−842)			(AD 842−1204)			(AD 1204−1430)			(AD 1430 − c. 1600)				
	*N*	%		*N*	%		*N*	%		*N*	%		*N*	%		*N*	%		*N*	%
Fetal (<birth)	0	0,0%		1	0,4%		0	0,0%		0	0,0%		0	0,0%		0	0,0%		1	0,2%
Infant (0–3 years)	2	3,7%		29	12,3%		1	1,3%		4	12,5%		4	5,8%		2	3,3%		42	7,9%
Child (4–12 years)	5	9,3%		56	23,8%		18	22,5%		3	9,4%		7	10,1%		11	18,0%		100	18,8%
Adolescent (13–18 years)	6	11,1%		16	6,8%		5	6,3%		2	6,3%		6	8,7%		8	13,1%		43	8,1%
Young adult (19–34 years)	16	29,6%		61	26,0%		30	37,5%		16	50,0%		27	39,1%		25	41,0%		175	33,0%
Middle adult (35–49 vears)	12	22,2%		53	22,6%		22	27,5%		6	18,8%		19	27,5%		15	21.6%		127	23,9%
Old Adult (50 + years)	13	24,1%		19	8,1%		4	5,0%		1	3,1%		6	8,7%		0	0,0%		43	8,1%
Total	54	100,0%		235	100,0%		80	100,0%		32	100,0%		69	100,0%		61	100,0%		531	100,0%
Nonadult (0–19)	0	0,0%		1	1,7%		1	3,7%		0	0,0%		0	0,0%		0	0,0%		2	1,5%
Adult (20 +years)	9	100,0%		57	98,3%		26	96,3%		12	100,0%		25	100,0%		2	100,0%		131	98.5%
Total	9	100,0%		58	100,0%		27	100,0%		12	100,0%		25	100,0%		2	100,0%		133	100,0%
**Total sample**	**63**			**293**			**107**			**44**			**94**			**63**			**664**	

**Table 2:** Results of Cox proportional hazard model for the covariates of sex, chronological period and topography of the burial (east and West cemetery)

	Hazard Ratio	SE	95% CI	z	*p*-value
Period	1.210	0.041	1.115–1.313	4.586	< 0.001
Cemetery	0.879	0.084	0.745–1.037	–1.530	0.126
Sex	1.076	0.109	0.869–1.334	0.677	0.498
Concordance = 0.575 (SE = 0.02)					
Likelihood ratio test = 21.57 on 3 df, *p* < 0.001					
Wald test = 22.63 on 3 df, *p* < 0.001					
Score (logrank) test=22.8 on 3 df, *p* < 0.001					

**Table 3:** The results of the Kaplan-Meier survival analysis by time period, with the mean and median survival times, their corresponding 95% confidence intervals and the results of the associated log-rank test

	*N*	Mean survival time (years)	SE	Median survival time (years)	Median survival time 95% CI
Hellenistic	57	39.623	2.359	37.500	32.500–47.500
Roman	208	39.495	1.193	37.500	32.500–42.500
Early Byzantine	91	38.269	1.739	37.500	32.500–42.500
Middle Byzantine	37	37.229	2.394	32.500	27.500–42.500
Late Byzantine	84	38.333	1.851	37.500	32.500–42.500
Post Byzantine	51	28.088	1.478	27.500	22.500–32.500
Log-rank test:	Chisq: 28.4 df: 5 *p* < 0.0001				

## Data Availability

This article and the SI include all data generated and analyzed during this study.
