# Supplementary material for: Urban survival, population dynamics and historical transitions in Thessaloniki (316 BC-AD 1500)
Source: Archaeol Anthropol Sci. Author manuscript; Available in PMC 2026 Aug 20. (PMC7619378; doi:10.1007/s12520-026-02439-z)
Supplement: Supplementary file 1 [file EMS217082-supplement-Supplementary_Information.docx]

# Urban survival, population dynamics and historical transitions in Thessaloniki (316 BC-AD 1500)

Asterios Aidonis^1,2^, Christina Kakasa^1,2^, Elissavet Ganiatsou^1,2^, Panagiota Bantavanou^1,2^, Soultana Protopsalti^3^, Stavroula Tzevreni^3^, Stela Vasileiadou^3^, Krino Konstantinidou^3^, and Christina Papageorgopoulou^1,2*^

*^1^Laboratory of Biological Anthropology, Department of Humanities, Democritus University of Thrace, 69100 Komotini, Greece,*

*^2^Institute of Applied Bioarchaeological Research, Democritus University of Thrace, Komotini, Greece*

*^3^ Ephorate of Antiquities of Thessaloniki City, Ministry of Culture, 54646 Thessaloniki, Greece*

** corresponding author: Christina Papageorgopoulou (cpapage@hs.duth.gr)*

# **Supplementary Information**

## **Nonadult representativeness**

**Table S1.** Estimation of indices evaluating the proportions of non-adult age groups in the skeletal sample, using the mortAAR package in R (Mueller-Scheessel et al., 2025)

|  | **Approach** | **Condition** | **Value1** | **Value2** | **Result** | **Outcome** |
| --- | --- | --- | --- | --- | --- | --- |
| **Hellenistic** | Weiss_1 | q_0-5_>q_15-20_ | 8.01 | 5.09 | 1.57 | TRUE |
|  | Weiss_2 | q_10-15_<q_15-20_ | 7.97 | 5.09 | 1.57 | FALSE |
|  | Bocquet-Apel_1 | (D_5-10_/D_10-15_)≥2 | 1.00 | 4.50 | 0.22 | FALSE |
|  | Bocquet-Apel_2 | (D_5-15_/D_20+_)≥0.1 | 5.50 | 49.29 | 0.11 | TRUE |
|  | TFR | TFR_SA = TFR_A | 3.59 | 8.35 | 4.76 | FALSE |
|  |  |  |  |  |  |  |
| **Roman** | Weiss_1 | q_0-5_>q_15-20_ | 19.15 | 4.12 | 4.64 | TRUE |
|  | Weiss_2 | q_10-15_<q_15-20_ | 5.30 | 4.12 | 1.29 | FALSE |
|  | Bocquet-Apel_1 | (D_5-10_/D_10-15_)≥2 | 29.08 | 10.95 | 2.66 | TRUE |
|  | Bocquet-Apel_2 | (D_5-15_/D_20+_)≥0.1 | 40.04 | 187.53 | 0.21 | TRUE |
|  | TFR | TFR_SA = TFR_A | 4.75 | 9.10 | 4.35 | FALSE |
|  |  |  |  |  |  |  |
| **Early Byzantine** | Weiss_1 | q_0-5_>q_15-20_ | 4.11 | 1.51 | 2.72 | TRUE |
|  | Weiss_2 | q_10-15_<q_15-20_ | 7.80 | 1.51 | 5.16 | FALSE |
|  | Bocquet-Apel_1 | (D_5-10_/D_10-15_)≥2 | 12.38 | 7.00 | 1.77 | FALSE |
|  | Bocquet-Apel_2 | (D_5-15_/D_20+_)≥0.1 | 19.38 | 81.44 | 0.24 | TRUE |
|  | TFR | TFR_SA = TFR_A | 3.99 | 9.42 | 5.43 | FALSE |
|  |  |  |  |  |  |  |
| **Middle Byzantine** | Weiss_1 | q_0-5_>q_15-20_ | 9.14 | 5.44 | 1.68 | TRUE |
|  | Weiss_2 | q_10-15_<q_15-20_ | 0.90 | 5.44 | 0.17 | TRUE |
|  | Bocquet-Apel_1 | (D_5-10_/D_10-15_)≥2 | 2.67 | 0.33 | 8.00 | TRUE |
|  | Bocquet-Apel_2 | (D_5-15_/D_20+_)≥0.1 | 3.00 | 34.77 | 0.09 | FALSE |
|  | TFR | TFR_SA = TFR_A | 3.53 | 9.55 | 6.01 | FALSE |
|  |  |  |  |  |  |  |
| **Late Byzantine** | Weiss_1 | q_0-5_>q_15-20_ | 6.42 | 4.53 | 1.42 | TRUE |
|  | Weiss_2 | q_10-15_<q_15-20_ | 4.28 | 4.53 | 0.94 | TRUE |
|  | Bocquet-Apel_1 | (D_5-10_/D_10-15_)≥2 | 3.80 | 3.58 | 1.06 | FALSE |
|  | Bocquet-Apel_2 | (D_5-15_/D_20+_)≥0.1 | 7.38 | 76.40 | 0.10 | FALSE |
|  | TFR | TFR_SA = TFR_A | 3.41 | 9.21 | 5.80 | FALSE |
|  |  |  |  |  |  |  |
| **Post Byzantine** | Weiss_1 | q_0-5_>q_15-20_ | 9.53 | 4.62 | 2.06 | TRUE |
|  | Weiss_2 | q_10-15_<q_15-20_ | 14.57 | 4.62 | 3.15 | FALSE |
|  | Bocquet-Apel_1 | (D_5-10_/D_10-15_)≥2 | 5.50 | 7.50 | 0.73 | FALSE |
|  | Bocquet-Apel_2 | (D_5-15_/D_20+_)≥0.1 | 13.00 | 41.94 | 0.31 | TRUE |
|  | TFR | TFR_SA = TFR_A | 4.56 | 10.96 | 6.40 | FALSE |
|  |  |  |  |  |  |  |
| **Total** | Weiss_1 | q_0-5_>q_15-20_ | 12.29 | 4.00 | 3.08 | TRUE |
|  | Weiss_2 | q_10-15_<q_15-20_ | 6.45 | 4.00 | 1.61 | FALSE |
|  | Bocquet-Apel_1 | (D_5-10_/D_10-15_)≥2 | 54.43 | 33.86 | 1.61 | FALSE |
|  | Bocquet-Apel_2 | (D_5-15_/D_20+_)≥0.1 | 88.29 | 471.36 | 0.19 | TRUE |
|  | TFR | TFR_SA = TFR_A | 4.23 | 9.29 | 5.06 | FALSE |

## **Preservation index**

After registering the presence of bone elements for each skeleton, the Individual Score (IS) was calculated. The Individual Score is the sum of the present elements divided by the total number of expected observations. It is always a positive number between 0 and 1. The average IS of the population is the Average Preservation Index (API). The API can also serve as a quantitative measure of preservation for age and sex subgroups of the sample (Stojanowski et al., 2002). The total preservation of the sample (API) calculated to 0.2681 (26.8%). Infants (IS=0.0831) and children (IS=0.0883) show significantly lower preservation (Kruskal Wallis p=0.00) (Table S2-3).

**Table S2.** Preservation of bones by age group as expressed by the mean of the Individual Score (IS) index.

| **Individual Score index** | | | |
| --- | --- | --- | --- |
| **Age** | **Mean** | **N** | **Std. Deviation** |
| Infant (b-3) | .0831 | 68 | .10470 |
| Child (3-12) | .0883 | 71 | .13502 |
| Adolescent (12-20) | .2580 | 25 | .22096 |
| Young Adult (20-35) | .3573 | 84 | .22540 |
| Middle Adult (35-50) | .4557 | 82 | .21665 |
| Old Adult (50+) | .3776 | 25 | .19143 |
| Total (API) | .2681 | 355 | .24100 |

**Table S3.** The results of the Kruskal Wallis test show that the difference in IS between age groups is statistically significant

| **Hypothesis Test Summary** | | | | |
| --- | --- | --- | --- | --- |
|  | **Null Hypothesis** | **Test** | **Sig.** | **Decision** |
| **1** | The distribution of IS is the same across categories of Age. | Independent-Samples Kruskal-Wallis Test | ,000 | Reject the null hypothesis. |
| Asymptotic significances are displayed. The significance level is ,05. | | | | |

## **Age-at-death distributions - life tables**

**Table S4.** Quotes from the life tables constructed with the mortAAR package for each chronological period. (dx) represents the proportion of the number of individuals of each period that fall within the corresponding age interval and (ex) life expectancy. Bold values represent life expectancy at birth (e0) and at twenty years (e20).

|  | **Hellenistic** | | **Roman** | | **Early Byzantine** | | **Middle Byzantine** | | **Late Byzantine** | | **Post Byzantine** | |
| --- | --- | --- | --- | --- | --- | --- | --- | --- | --- | --- | --- | --- |
| **x** | **dx** | **ex** | **dx** | **ex** | **dx** | **ex** | **dx** | **ex** | **dx** | **ex** | **dx** | **ex** |
| 0-4 | 8.01 | **36.82** | 19.15 | **28.96** | 4,11 | **32,83** | 9,14 | **32,78** | 6,42 | **34,99** | 9.53 | **24.61** |
| 5-9 | 1.60 | 34.88 | 9.98 | 30.42 | 11,63 | 29,17 | 6,09 | 30,90 | 4,07 | 32,28 | 8.74 | 22.03 |
| 10-14 | 7.21 | 30.46 | 3.76 | 29.35 | 6,58 | 27,85 | 0,76 | 27,95 | 3,83 | 28,63 | 11.91 | 19.12 |
| 15-19 | 4.24 | 27.88 | 2.77 | 25.85 | 1,17 | 24,99 | 4,57 | 23,18 | 3,88 | 24,80 | 3.23 | 16.95 |
| 20-24 | 15.04 | **24.24** | 11.01 | **21.86** | 10,46 | **20,34** | 5,86 | **19,37** | 14,69 | **20,85** | 19.84 | **12.65** |
| 25-29 | 7.04 | 24.36 | 6.84 | 20.86 | 13,75 | 18,16 | 19,29 | 15,71 | 10,58 | 19,87 | 12.70 | 11.96 |
| 30-34 | 7.04 | 22.07 | 8.13 | 18.56 | 9,99 | 17,28 | 18,14 | 15,40 | 10,22 | 18,12 | 9.92 | 10.49 |
| 35-39 | 7.04 | 19.83 | 7.08 | 16.96 | 8,73 | 15,76 | 7,37 | 16,88 | 9,87 | 16,57 | 8.06 | 8.77 |
| 40-44 | 7.84 | 17.68 | 7.65 | 15.22 | 9,67 | 14,21 | 8,51 | 15,57 | 10,40 | 15,38 | 7.27 | 6.91 |
| 45-49 | 7.84 | 16.08 | 7.65 | 14.34 | 8,27 | 13,95 | 4,57 | 16,06 | 6,66 | 15,53 | 7.27 | 5.55 |
| 50-54 | 4.52 | 15.00 | 2.67 | 15.00 | 2,61 | 15,00 | 2,62 | 15,00 | 3,23 | 15,00 | 0.26 | 15.00 |
| 55-59 | 4.52 | 12.50 | 2.67 | 12.50 | 2,61 | 12,50 | 2,62 | 12,50 | 3,23 | 12,50 | 0.26 | 12.50 |
| 60-64 | 4.52 | 10.00 | 2.67 | 10.00 | 2,61 | 10,00 | 2,62 | 10,00 | 3,23 | 10,00 | 0.26 | 10.00 |
| 65-69 | 4.52 | 7.50 | 2.67 | 7.50 | 2,61 | 7,50 | 2,62 | 7,50 | 3,23 | 7,50 | 0.26 | 7.50 |
| 70-74 | 4.52 | 5.00 | 2.67 | 5.00 | 2,61 | 5,00 | 2,62 | 5,00 | 3,23 | 5,00 | 0.26 | 5.00 |
| 75-79 | 4.52 | 2.50 | 2.67 | 2.50 | 2,61 | 2,50 | 2,62 | 2,50 | 3,23 | 2,50 | 0.26 | 2.50 |

Life expectancy at birth (e0) shows fluctuations between the periods under study, ranging from 36.82 years in the Hellenistic period to 24.61 years to the Post Byzantine sample. These differences are primarily attributed to the differential representation of infants in the chronological samples. The life expectancy at twenty (e20) presents a gradual decline from the Hellenistic (24,4 years) to the Post Byzantine period, where the lowest value is observed (12.65 years).

**Table S5.** The results of the Mann-Witney U test, examining the age-at-death distributions across the specified periods.

| **Period** | **N** | **Mean Rank** | **p-value** |
| --- | --- | --- | --- |
| Hellenistic | 57 | 133.58 | 0.948 |
| Roman | 208 | 132.84 |  |
| Roman | 208 | 151.56 | 0.635 |
| Early Byzantine | 91 | 146.43 |  |
| Early Byzantine | 91 | 65.16 | 0.751 |
| Middle Byzantine | 37 | 62.88 |  |
| Middle Byzantine | 37 | 59.74 | 0.792 |
| Late Byzantine | 84 | 61.55 |  |
| Late Byzantine | 84 | 76.98 | 0.001 |
| Post Byzantine | 51 | 53.21 |  |

## **Sex ratio**

**Table S6.** The estimated sex ratio by chronological period.

| **Period** | **Males** | **Females** | **Sex Ratio** |
| --- | --- | --- | --- |
| Hellenistic | 19_a_ | 27_a_ | 70,4 |
| Roman | 64_a_ | 72_a_ | 88,9 |
| Early Byzantine | 24_a_ | 35_a_ | 68,6 |
| Middle Byzantine | 10 _a_ | 19 _a_ | 52,6 |
| Late Byzantine | 30 _a_ | 22 _a_ | 136,4 |
| Post Byzantine | 29_a_ | 12_b_ | 241.7 |
| *Each subscript letter denotes a subset of Sex categories whose column proportions do not differ significantly from each other at the ,05 level.* | | | |
| Pearson Chi-Square: |  |  |  |
| Value=14.666 |  |  |  |
| df=5 |  |  |  |
| p=0.012 |  |  |  |

## **Survival analysis**

**Table S7.** The results of the test for the proportional hazards assumption of the Cox analysis.

|  | **chisq** | **df** | **p-value** |
| --- | --- | --- | --- |
| Period | 0.5185 | 1 | 0.47 |
| Cemetery | 0.8718 | 1 | 0.35 |
| Sex | 0.0522 | 1 | 0.82 |
| Global | 0.9796 | 3 | 0.81 |

**Table S8.** The results of the Kaplan-Meier survival analysis by sex in each chronological period, with the mean and median survival times, their corresponding 95% confidence intervals and the results of the associated log-rank test.

|  | **Females** | | | | | **Males** | | | | |  |  |
| --- | --- | --- | --- | --- | --- | --- | --- | --- | --- | --- | --- | --- |
| **Period** | **N** | **Mean**  **survival time**  **(years)** | **SE** | **Median survival time (years)** | **Median survival time 95% CI** | **N** | **Mean**  **survival time**  **(years)** | **SE** | **Median survival time (years)** | **Median survival time 95% CI** | **Chisq** | **p-value** |
| Hellenistic | 26 | 43.846 | 3.403 | 42.500 | 32.500-57.500 | 19 | 43.552 | 3.896 | 42.500 | 37.500-62.500 | 0.000 | 0.990 |
| Roman | 70 | 39.785 | 1.742 | 37.500 | 32.500-42.500 | 63 | 42.658 | 1.995 | 42.500 | 37.500-47.500 | 1.000 | 0.320 |
| Early Byzantine | 35 | 37.928 | 2.452 | 32.500 | 27.500-42.500 | 24 | 39.375 | 2.838 | 37.500 | 32.500-47.500 | 0.200 | 0.650 |
| Middle Byzantine | 21 | 42.023 | 3.577 | 37.500 | 32.500-57.500 | 10 | 38.000 | 4.150 | 32.500 | 27.500-NA | 0.300 | 0.560 |
| Late Byzantine | 21 | 37.738 | 3.185 | 32.500 | 27.500-47.500 | 32 | 39.687 | 2.585 | 37.500 | 32.500-42.500 | 0.100 | 0.720 |
| Post Byzantine | 12 | 30.000 | 2.394 | 27.500 | 22.500-NA | 28 | 31.964 | 1.603 | 30.000 | 27.500-500 | 0.300 | 0.560 |

**Table S 9.** The results of the Kaplan-Meier survival analysis by cemetery in each chronological period, with the mean and median survival times, their corresponding 95% confidence intervals and the results of the associated log-rank test.

|  | **West cemetery** | | | | | **East cemetery** | | | | | **Intramural** | | | | |  |  |
| --- | --- | --- | --- | --- | --- | --- | --- | --- | --- | --- | --- | --- | --- | --- | --- | --- | --- |
| **Period** | **N** | **Mean**  **survival time**  **(years)** | **SE** | **Median survival time (years)** | **Median survival time 95% CI** | **N** | **Mean**  **survival time**  **(years)** | **SE** | **Median survival time (years)** | **Median survival time 95% CI** | **N** | **Mean**  **survival time**  **(years)** | **SE** | **Median survival time (years)** | **Median survival time 95% CI** | **Chisq** | **p-value** |
| Hellenistic | 45 | 39.633 | 2.654 | 37.500 | 27.500 -47.500 | 12 | 39.583 | 5.152 | 40.000 | 27.500-NA | - | - | - | - | - | 0.000 | 0.880 |
| Roman | 81 | 39.105 | 1.922 | 37.500 | 32.500-42.500 | 127 | 39.744 | 1.521 | 37.500 | 32.500-42.500 | - | - | - | - | - | 0.000 | 0.840 |
| Early Byzantine | 38 | 33.947 | 2.467 | 32.500 | 27.500- 42.500 | 19 | 43.815 | 3.864 | 42.500 | 32.500- 62.500 | 33 | 37.803 | 2.801 | 32.500 | 27.500- 42.500 | 3.200 | 0.200 |
| Middle Byzantine | - | - | - | - | - | 2 | 35.000 | 5.303 | 35.000 | 27.500-NA | 35 | 36.357 | 2.587 | 32.500 | 27.500- 37.500 | 0.000 | 0.910 |
| Late Byzantine | 5 | 27.500 | 3.741 | 27.500 | 22.500- NA | 1 | 27.500 | 0.000 | 27.500 | NA -NA | 78 | 39.230 | 1.941 | 37.500 | 32.500- 42.500 | 4.5 | 0.110 |
| Post Byzantine | 48 | 27.604 | 1.527 | 27.500 | 22.500-32.500 | 3 | 35.833 | 3.600 | 37.500 | 27.500-NA |  |  |  |  |  | 0.600 | 0.440 |

**Table S10.** D_5-19_/D_5+_ with its 95 % comparison intervals (CI), the Crude Birth Rate (CBR), the Growth Rate (r) estimated according to Bocquet‐Appel (2002), and the r* indicator according to Barbiera et al. (2018).

|  | **D_5-19_/D_5+_** | **Lower 95% CI** | **Upper 95% CI** | **Growth Rate (r)** | **r*** |
| --- | --- | --- | --- | --- | --- |
| Hellenistic | 0,1379 | 0,0034 | 0,2724 | -0,005 | 0,1604 |
| Roman | 0,2017 | 0,1353 | 0,2681 | 0,005 | 0,1792 |
| Early Byzantine | 0,2039 | 0,1030 | 0,3048 | 0,005 | 0,1814 |
| Middle Byzantine | 0,1250 | -0,0369 | 0,2869 | -0,007 | 0,1565 |
| Late Byzantine | 0,1250 | 0,0158 | 0,2342 | -0,007 | 0,1565 |
| Post Byzantine | 0,2632 | 0,1275 | 0,3988 | 0,012 | 0,2092 |
